# Intake and sources of dietary fibre and dietary fibre fractions in Finnish children

**DOI:** 10.1017/S0007114523000466

**Published:** 2023-10-28

**Authors:** Tuuli E. I. Salo, Sari Niinistö, Tuuli E. Korhonen, Helena Pastell, Heli Reinivuo, Hanna-Mari Takkinen, Jorma Ilonen, Jorma Toppari, Mikael Knip, Riitta Veijola, Suvi M. Virtanen

**Affiliations:** 1 Department of Public Health and Welfare, Finnish Institute for Health and Welfare, FI-00271 Helsinki, Finland; 2 Unit of Health Sciences, Faculty of Social Sciences, Tampere University, FI-33014 Tampere, Finland; 3 Finnish Food Authority, Mustialankatu 3, FI-00790 Helsinki, Finland; 4 Research, Development and Innovation Center, Tampere University Hospital, P.O. Box 2000, FI-33521 Tampere, Finland; 5 Immunogenetics Laboratory, Institute of Biomedicine, University of Turku, FI-20014 Turku, Finland; 6 Research Centre for Integrative Physiology and Pharmacology, Institute of Biomedicine, University of Turku, FI-20520 Turku, Finland; 7 Department of Pediatrics, Turku University Hospital, FI-20520 Turku, Finland; 8 Pediatric Research Center, Children’s Hospital, University of Helsinki and Helsinki University Hospital, FI-00029 Helsinki, Finland; 9 Research Program for Clinical and Molecular Metabolism, Faculty of Medicine, University of Helsinki, FI-00014 Helsinki, Finland; 10 Department of Pediatrics, Tampere University Hospital, FI-33521 Tampere, Finland; 11 Department of Pediatrics, PEDEGO Research Unit, Medical Research Center Oulu, University of Oulu and Oulu University Hospital, FI-90014 Oulu, Finland; 12 Department of Children and Adolescents, Oulu University Hospital, P.O. Box 10, FI-90029 Oulu, Finland; 13 Center for Child Health Research, Tampere University and Tampere University Hospital, FI-33014 Tampere, Finland

**Keywords:** Dietary fibre, Dietary fibre fractions, Food composition database, Children, Intake, Human milk oligosaccharides

## Abstract

The current definition of dietary fibre was adopted by the Codex Alimentarius Commission in 2009, but implementation requires updating food composition databases with values based on appropriate analysis methods. Previous data on population intakes of dietary fibre fractions are sparse. We studied the intake and sources of total dietary fibre (TDF) and dietary fibre fractions insoluble dietary fibre (IDF), dietary fibre soluble in water but insoluble in 76 % aqueous ethanol (SDFP) and dietary fibre soluble in water and soluble in 76 % aqueous ethanol (SDFS) in Finnish children based on new CODEX-compliant values of the Finnish National Food Composition Database Fineli. Our sample included 5193 children at increased genetic risk of type 1 diabetes from the Type 1 Diabetes Prediction and Prevention birth cohort, born between 1996 and 2004. We assessed the intake and sources based on 3-day food records collected at the ages of 6 months, 1, 3 and 6 years. Both absolute and energy-adjusted intakes of TDF were associated with age, sex and breast-feeding status of the child. Children of older parents, parents with a higher level of education, non-smoking mothers and children with no older siblings had higher energy-adjusted TDF intake. IDF was the major dietary fibre fraction in non-breastfed children, followed by SDFP and SDFS. Cereal products, fruits and berries, potatoes and vegetables were major food sources of dietary fibre. Breast milk was a major source of dietary fibre in 6-month-olds due to its human milk oligosaccharide content and resulted in high SDFS intakes in breastfed children.

Higher intake of dietary fibre is associated with several health benefits, including but not limited to reduced risk of all-cause mortality, CVD, type 2 diabetes, obesity and colorectal cancer^(1–4)^. Higher intake of dietary fibre can also be considered a marker of an overall healthy diet, as dietary fibre is most abundant in minimally processed plant-based foods such as whole grains, fruits, vegetables, legumes, nuts and seeds^(5,6)^. Dietary fibre differs from other nutrients in that it is an umbrella term for a diverse group of different compounds. The individual types of dietary fibre vary in their physicochemical and functional characteristics such as chain length, types of polymer linkages, solubility, viscosity and fermentability, resulting in differing health effects during passage through the gastro-intestinal tract^(6,7)^. Indeed, individual dietary fibre types have differing health claims authorised by the European Food Safety Authority, including reduction of the blood glucose rise after a meal, increase in faecal bulk, contribution to normal bowel function, contributions to an acceleration of intestinal transit, maintenance of normal blood cholesterol levels and contribution to weight loss in the context of an energy-restricted diet^(8)^.

The history of studying dietary fibre has been complicated by changing definitions and analysis methods. As a results, certain types of dietary fibre have been either missed due to use of older methods that cannot measure all components or overestimated due to use of several methods that partially measure the same fibres twice^(9,10)^. The internationally approved CODEX definition for dietary fibre was finally adopted in June 2009 following 16 years of debate^(11,12)^. The definition includes previously poorly represented types of dietary fibre such as resistant starch, oligosaccharides, polydextrose and resistant maltodextrins^(9)^. The choice of including non-digestible oligosaccharides (NDO), which consist of less than ten monomeric units, has been left up to national decision making, but so far, most countries have opted to include them^(13,14)^.

Previously developed analysis methods have not been able to analyse all different dietary fibres according to the new definition. The enzymatic-gravimetric-liquid chromatographic AOAC Official method 2009·01 and its extension 2011·25 have been developed to match the new CODEX definition of dietary fibre^(15,16)^. The Finnish National Food Composition Database Fineli is among the first to be updated with new values since the establishment of the CODEX definition. The new data include total dietary fibre values as well as dietary fibre fractions matching the AOAC 2011·25 method: insoluble dietary fibre (IDF), soluble dietary fibre that precipitates in 76 % aqueous ethanol (SDFP; referred to as high-molecular-weight-soluble dietary fibre in earlier AOAC methods^(17,18)^) and soluble dietary fibre that remains soluble in 76 % aqueous ethanol (SDFS; referred to as low-molecular-weight dietary fibre or NDO in earlier AOAC methods^(17,18)^).

There are minimal previous data on population intakes of the different dietary fibre fractions. Furthermore, nutritional epidemiology requires up to date analysis methods and food composition databases to minimise systematic error sources. The purpose of this study is to assess the information gap by providing novel information on the intake and sources of dietary fibre and dietary fibre fractions in Finnish children, as well as observe differences between socio-demographic groups in dietary fibre intake.

## Methods

### Study population

The participants of the current study were sampled from the Type 1 Diabetes Prediction and Prevention (DIPP) Nutrition Study. The DIPP Nutrition Study was conducted as part of the main DIPP Study, which is a population-based birth cohort of Finnish children with HLA-conferred susceptibility to type 1 diabetes assessed from cord blood samples. All children with either medium or high susceptibility to type 1 diabetes born in Oulu University Hospital between September 1996 and September 2004, or Tampere University Hospital between 1997 and 2004 were invited to take part in the DIPP and DIPP Nutrition Studies. Participants of the DIPP Nutrition Study had information on dietary intake collected with 3-day food records until the age of 6 years in addition to the standard processes of the main DIPP Study. Information on sociodemographic factors was collected after the delivery with a structured questionnaire filled out by the parents.

This study assessed children’s dietary fibre intake at the ages of 6 months, 1, 3 and 6 years. All children in the DIPP Nutrition Study with at least one complete 3-day food record at any of the studied age points were included in the sample. 6-month-olds and 1-year-olds with missing growth information necessary for breast milk intake estimation were excluded at these age points. Children who developed autoimmunity were included.

### Assessment of dietary intake

Three-day food records were collected at all the studied age points. The process of collecting the food records has been described in detail earlier^(19)^. Open-ended food records with detailed instructions and pre-set dates of three consecutive days, of which 2 weekdays and 1 weekend day were provided, with separate copies for the caretakers and for day care personnel. Household measures, package sizes and weights when available were used for portion size estimation. All food records were checked upon return by trained research nurses. Dietary data were handled by trained research nutritionists. The food record data were entered into Finessi, an in-house dietary calculation software of the Finnish Institute for Health and Welfare. Finessi utilises data from the Finnish National Food Composition Database Fineli, which is maintained and updated annually by the Finnish Institute for Health and Welfare.

The database Fineli includes both food ingredients, e.g. rolled oats, as well as dishes compiled from ingredients via individual recipes, e.g. oatmeal, which includes the ingredients rolled oats, iodised salt, and 1·5 % fat milk with added vitamin D. The ingredient information for dishes is commonly based on ingredient and nutrition values declared by the manufacturer for commercial foods, and information from commonly used cookery books in Finland for non-commercial foods. Nutrient values for dishes are further based on the comprehensive nutrient values of the individual ingredients. The database also takes into account the physicochemical effects the cooking process has on the nutritional values of the dishes. There are roughly 2000 ingredients and over 3000 compiled dishes in Fineli, depending on the version. Different versions of the database are used simultaneously for cohort data such as this study, since processing methods, ingredients of commercial foods, food fortification levels and products available on the market are time dependent, and the dietary data are collected over several years. Each food record was processed with a version of the database matching the time of collection.

Because of the structure of the database, and the fact that the dietary calculation software Finessi in linked with Fineli, all dietary data inserted into Finessi can be broken down into food ingredients. Furthermore, all food ingredients belong to an ingredient class, for example spinach and lettuce into the ingredient class ‘leaf vegetables’. Breaking all foods down into ingredients and summarising them according to ingredient classes (for example, combining all ingredients from ingredient classes ‘leaf vegetables’, ‘root vegetables’ and ‘fruit vegetables’ as well as other desired vegetable classes into the food source classification ‘vegetables’) enables carrying out *source analysis*, in which the intake of a nutrient deriving from different food sources is assessed.

In our study, we assessed the food sources of total dietary fibre as well as dietary fibre fractions according to the following food source classifications:

1. Wheat
2. Rye
3. Oat
4. Other cereal products (barley, rice and other cereals)
5. Fruits and berries (includes juices)
6. Vegetables (includes mushrooms)
7. Potatoes
8. Legumes, nuts and seeds
9. Formula
10. Others (all ingredients not included above: meats, dairy products, fats, condiments, sweets, etc. May include some fibre sources, e.g. chocolate, ketchup.)

In this study, breast-feeding status was determined in 6-month-olds and 1-year-olds based on whether they had received any breast milk on the recorded 3 days. Caretakers primarily recorded the frequency of breast-feeding, not the amount of breast milk consumed. The amount of breast milk was calculated retrospectively using estimated energy requirements based on child weight and the growth of the child, and the energy intake from other foods based on the food records^(20)^. Energy and dietary fibre from breast milk were included in energy and dietary fibre intake for 6-month-olds and 1-year-olds. After the age of 1 year breast milk intake was not considered due to very infrequent and little use by older children. We did not include dietary fibre supplements.

### Compiling of dietary fibre values

The Finnish Food Authority and the Finnish Institute for Health and Welfare were responsible for the process of updating dietary fibre values of Fineli. As mentioned previously, Fineli consists of both food ingredients and dishes. Since dishes are compiled from food ingredients, it was only necessary to produce updated dietary fibre values for food ingredients. Updating the values for food ingredients would allow recalculation of the values for dishes as well.

The Finnish Food Authority conducted novel dietary fibre analyses for 110 food ingredients with the AOAC 2011·25 method, which can separately analyse IDF, SDFP, and SDFS (TDF = IDF + SDFP + SDFS)^(21,22)^. Values for additional ninety-six food ingredients were extracted from previous literature. Of these literature-based values, eighteen were based on AOAC 2009·01 (which separates HMWDF (IDF combined with SDFP), and SDFS) or AOAC 2011·twenty-five analyses, thirty-seven on AOAC 991·forty-three analyses (HMWDF, underestimates resistant starch, inulin and resistant maltodextrins) and forty one on other methods. After the analyses and literature review, values for 134 food ingredients were imputed from related foods. Values for additional thirty-nine food ingredients were derived from previous analyses carried out by the Finnish Food Authority. Finally, values for 1839 food ingredients could be calculated based on all the above and the recipes of the individual food ingredients. Not only dishes include recipes in Fineli, but food ingredients too, e.g. apple jam, which is at the same time a food ingredient, and has a recipe consisting of sugar, ‘water, tap water’ and ‘apple, average, with skin’.

Dietary fibre content of breast milk was based on previous literature on human milk oligosaccharides (HMO)^(23)^. Although HMO secretion into breast milk varies between individuals as well as over the period of lactation, we estimated 100 grams of breast milk to contain 1·3 grams of HMO, which is a value previously reported for mature milk (120+ days of lactation)^(23)^. Dietary fibre in breast milk was inserted into Fineli as SDFS since HMO are NDO.

### Statistical methods

Dietary fibre intake was analysed both in grams per day as well as energy-adjusted for grams per megajoule. Medians and interquartile ranges were calculated to describe the intake of dietary fibre in different subgroups of the sample. Pearson’s chi-square test was used to test for differences in the frequencies of background variables between the age groups in order to assess differences in drop-out rate.

Age differences in absolute and energy-adjusted intake of total dietary fibre as well as dietary fibre fractions were tested separately for girls and boys. Sex differences in dietary fibre intake were tested separately according to age group and possible breastfeeding status subgroup within the age group. Differences in dietary fibre intake associated with breast-feeding status were tested separately according to age group and sex.

Age differences were assessed using univariate analysis of variance. Tukey’s honest significance difference post hoc test was used to determine which age groups differed in the age group tests. Sex and breastfeeding status differences were assessed with independent samples *t* test and the non-parametric Mann–Whitney U-Test as appropriate for data distribution. Data was normally distributed for all dietary fibre fractions in 3-year-olds and 6-year-olds but skewed for all fractions in 6-month-olds and SDFS in 1-year-olds.

Differences in energy-adjusted intake of total dietary fibre according to socio-demographic factors in 1-, 3-, and 6-year-olds were assessed using independent samples *t* test for binary variables, and univariate analysis of variance for non-binary variables. Breastfed 1-year-olds were excluded from the analysis to reduce within-group variance. Food sources of total dietary fibre and dietary fibre fractions were determined by source analysis, utilising the ingredient level dietary data as described previously. Statistical significance was predetermined as *P*-values ≤ 0·05. Multiple testing was controlled for using the false discovery rate (FDR) method^(24)^. Statistical analyses were carried out in IBM SPSS Statistics 27.

### Ethical considerations

Informed consent is required for the genetic screening of the DIPP study. Parents of the DIPP study children have had high participation incentive. The ethics committees of all the universities and university hospitals participating in the DIPP study have approved the study.

The current study was observational and utilised already existing data from the DIPP Nutrition Study. Carrying out the study did not cause additional participation burden for the study participants but instead allowed the high-quality data that was produced with the combined effort of the participants and research staff to be analysed as exhaustively as possible.

## Results

A total of 5193 children were included in the sample. The sub-sample sizes at each age point as well as other characteristics of the sample are presented in Table 1. Children born in the Oulu University Hospital region (*P* < 0·001), children of younger mothers (*P* = 0·003), children of less highly educated mothers (*P* = 0·048), and children of mothers who smoked during the pregnancy (*P* = 0·018) had higher drop-out rates. After correction for multiple testing with the FDR method however, only the region and mother’s age remained significant factors for drop-out rates (Table 1). At the age of 6 months, 58·5 % of the children were breastfed, while at the age of 1 year, only 18·4 % of the children were breastfed.

**Table 1.** Characteristics of the 5193 study participants

	Age group								
	6 months		1 year		3 years		6 years		
	*n*	%	*n*	%	*n*	%	*n*	%	*P* value*
Sex									0·897
Girl	2278	47	2051	46	1635	47	1106	47	
Boy	2608	53	2365	54	1828	53	1243	53	
Region									< 0·001†
Tampere University Hospital	2723	56	2472	56	1986	57	1445	62	
Oulu University Hospital	2163	44	1944	44	1477	43	904	39	
Mother’s age (years)									0·003†
< 25	898	18	755	17	542	16	336	14	
25–29	1701	35	1577	36	1236	36	840	36	
30–34	1451	30	1317	30	1053	30	722	31	
≥ 35	836	17	767	17	632	18	451	19	
Father’s age (years)									0·054
< 25	476	10	405	9	302	9	170	7	
25–29	1407	29	1272	29	988	29	659	28	
30–34	1570	32	1434	33	1131	33	811	35	
≥ 35	1235	25	1126	26	918	27	624	27	
Missing	198	4	179	4	124	4	85	4	
Mother’s education									0·048
Basic	277	6	231	5	160	5	90	4	
Vocational	1273	26	1138	26	873	25	588	25	
Secondary vocational	2145	44	1949	44	1553	45	1082	46	
Academic	1080	22	998	23	810	23	551	24	
Missing	111	2	100	2	67	2	38	2	
Father’s education									0·447
Basic	277	6	245	6	173	5	107	5	
Vocational	2015	41	1797	41	1407	41	953	41	
Secondary vocational	1356	28	1244	28	996	29	705	30	
Academic	1043	21	950	22	773	22	512	22	
Missing	195	4	180	4	114	3	72	3	
Highest education of the parents									0·571
Basic	58	1	51	1	33	1	22	1	
Vocational	1211	25	1070	24	808	23	537	23	
Secondary vocational	1987	41	1815	41	1428	41	985	42	
Academic	1540	32	1401	32	1143	33	774	33	
Missing	90	2	79	2	51	2	31	1	
Number of siblings									0·711
0	2346	48	2107	48	1664	48	1120	48	
1	1467	30	1338	30	1081	31	750	32	
≥ 2	958	20	870	20	654	19	439	19	
Missing	115	2	101	2	64	2	40	2	
Mother’s smoking during pregnancy									0·018
Non-smoking	4284	88	3894	88	3070	89	2094	89	
Smoking	445	9	387	9	283	8	163	7	
Missing	157	3	135	3	110	3	92	4	
Total	4886	100	4416	100	3463	100	2349	100	

* Pearson’s *χ*^2^ test.

† Statistically significant on ≤ 0·05 level after correction for multiple testing with false discovery rate method.

### Average intake of dietary fibre and dietary fibre fractions

Medians and interquartile ranges for absolute and energy-adjusted intake of total dietary as well as dietary fibre fractions are presented in Table 2.

**Table 2.** Absolute and energy-adjusted intakes of total dietary fibre (TDF), insoluble dietary fibre (IDF), dietary fibre soluble in water but insoluble in 76 % aqueous ethanol (SDFP) and dietary fibre soluble in water and soluble in 76 % aqueous ethanol (SDFS) in boys and girls by age and breast-feeding status

Girls													
		Age group											
		6 months				1 year				3 years		6 years	
		Breastfed		Not breastfed		Breastfed		Not breastfed		All		All	
		*n* 1356		*n* 922		*n* 394		*n* 1657		*n* 1635		*n* 1106	
		Median	IQR	Median	IQR	Median	IQR	Median	IQR	Median	IQR	Median	IQR
TDF	g/d	12·1	3·7	4·7	3	13·4	4·8	9·7	4·4	11·5	5	13·6	5·4
	g/MJ	4·58	0·99	1·64	0·97	3·96	1·25	2·68	1·09	2·32	0·9	2·3	0·87
IDF	g/d	2·3	2·1	2·9	1·9	5·8	3·2	5·8	2·7	6·6	3·3	7·9	3·5
	g/MJ	0·87	0·8	1·02	0·6	1·7	0·94	1·59	0·68	1·33	0·59	1·3	0·57
SDFP	g/d	1·2	1·1	1·5	1	2·9	1·8	3	1·5	3·3	1·4	3·8	1·5
	g/MJ	0·44	0·41	0·53	0·32	0·87	0·51	0·83	0·38	0·67	0·24	0·65	0·22
SDFS	g/d	8·16	5·18	0·23	0·26	3·65	4·67	0·85	0·62	1·3	0·83	1·7	0·98
	g/MJ	3·09	1·74	0·08	0·09	1·12	1·53	0·23	0·16	0·26	0·15	0·29	0·16

Boys													
		Age group											
		6 months				1 year				3 years		6 years	
		Breastfed		Not breastfed		Breastfed		Not breastfed		All		All	
		*n* 1504		*n* 1104		*n* 417		*n* 1948		*n* 1828		*n* 1243	
		Median	IQR	Median	IQR	Median	IQR	Median	IQR	Median	IQR	Median	IQR
TDF	g/d	13·1	4·2	5·2	3·4	14·5	4·7	10·8	5·2	12·2	5·7	14·8	6·3
	g/MJ	4·58	1·12	1·66	1	4·06	1·38	2·77	1·13	2·34	0·94	2·21	0·86
IDF	g/d	2·7	2·3	3·2	2·2	6·4	3·4	6·4	3·2	6·9	3·6	8·3	4·1
	g/MJ	0·94	0·78	1·02	0·63	1·76	0·94	1·62	0·71	1·32	0·59	1·24	0·57
SDFP	g/d	1·4	1·3	1·7	1·1	3·2	1·8	3·3	1·7	3·6	1·5	4·2	1·7
	g/MJ	0·48	0·42	0·54	0·32	0·9	0·48	0·86	0·39	0·68	0·25	0·64	0·21
SDFS	g/d	8·25	5·41	0·25	0·3	3·85	5·08	0·93	0·73	1·42	0·94	1·84	1·09
	g/MJ	2·96	1·69	0·08	0·09	1·06	1·47	0·24	0·18	0·27	0·16	0·28	0·16

### Differences in total dietary fibre intake according to age, sex, and breastfeeding status

Absolute intake of dietary fibre increased with age in both boys and girls (boys *P* < 0·001; girls *P* < 0·001), while energy-adjusted intake of total dietary fibre decreased with age (boys *P* < 0·001; girls *P* < 0·001). The post-hoc tests indicated that differences in energy-adjusted intake of total dietary fibre were not significant between 3- and 6-year-olds (boys 2·39 *v*. 2·30, *P* = 0·063, girls 2·37 g/MJ *v*. 2·34, *P =* 0·920). If breastfed and non-breastfed girls and boys were assessed separately, the peak in energy-adjusted dietary fibre intake occurred later, at the age of 1 year in non-breastfed children, as their intakes at the age of 6 months were considerably lower than those of breastfed counterparts.

Boys had higher intake of absolute total dietary fibre than girls at all age points within matched breast-feeding status subgroup (*P* < 0·001 for all subgroups). For energy-adjusted values, higher intake in boys was observed in non-breast-fed 1-year-olds (boys 2·82 g/MJ *v*. girls 2·73 g/MJ, *P* = 0·001), but there were no sex differences in breastfed 6-month-olds (*P* = 0·842), non-breastfed 6-month-olds (*P* = 0·428), breastfed 1-year-olds (*P* = 0·311), 3-year-olds (*P* = 0·245) or 6-year-olds (*P* = 0·151).

Breastfeeding status was associated with absolute intake of dietary fibre at the age of 6 months and 1 year. Breastfed boys and girls had higher absolute intakes at both age points than their non-breastfed counterparts, mostly due to considerably higher SDFS intake from breast milk (*P* < 0·001 in both sexes at both age points). This also translated into considerably higher energy-adjusted total dietary fibre intakes in both breastfed boys and girls at the ages of 6 months and 1 year (*P* < 0·001 in both sexes at both age points). Age, sex, and breastfeeding status differences in dietary fibre intake were unaffected by correction for multiple testing with the FDR method.

### Proportions of dietary fibre fractions

The proportions of the different dietary fibre fractions in relation to total dietary fibre are presented in Table 3. Proportions were similar for non-breastfed 1-year-olds, 3-year-olds, and 6-year-olds with IDF as the major fraction, followed by SDFP and SDFS. For breastfed children however, the proportion of SDFS was distinctly higher, accounting for over 65 % of dietary fibre in 6-month-old boys and girls, and over 26 % in 1-year-olds.

**Table 3.** Percentages of dietary fibre fractions out of total dietary fibre: insoluble dietary fibre (IDF), dietary fibre soluble in water but insoluble in 76 % aqueous ethanol (SDFP) and dietary fibre soluble in water and soluble in 76 % aqueous ethanol (SDFS) of total dietary fibre in boys and girls by age and breast-feeding status

Girls						
	Age group					
	6 months		1 year		3 years	6 years
	Breastfed	Not breastfed	Breastfed	Not breastfed	All	All
	*n* 1356	*n* 922	*n* 394	*n* 1657	*n* 1635	*n* 1106
	Median	Median	Median	Median	Median	Median
IDF, %	21·1	61·7	46·3	58·9	57·7	57·4
SDFP, %	11	32·8	23·2	31·1	29·1	28·2
SDFS, %	67·9	4·9	29	9·1	11·5	12·5

Boys						
	Age group					
	6 months		1 year		3 years	6 years
	Breastfed	Not breastfed	Breastfed	Not breastfed	All	All
	*n* 1504	*n* 1103	*n* 417	*n* 1948	*n* 1828	*n* 1243
	Median	Median	Median	Median	Median	Median
IDF, %	22·8	61·3	48·1	58·7	57·1	56·1
SDFP, %	11·7	32·9	23·7	31·4	29·5	28·9
SDFS, %	65·3	5	26·7	8·9	11·8	12·8

### Socio-demographic differences

Differences in energy-adjusted total dietary fibre intake by socio-demographic factors are presented in Table 4. Significant differences in intake were observed for all socio-demographic factors. However, the differences were not always significant at all age points. Generally, children of older, more highly educated parents, those with no older siblings and those with non-smoking mothers had higher energy-adjusted intake of total dietary fibre. Socio-demographic differences in dietary fibre intake were unaffected by correction for multiple testing with the FDR method. Intakes of energy-adjusted dietary fibre fractions were highly correlated with TDF as well as each other, and socio-demographic differences in fraction intakes reflected those of TDF (data not shown).

**Table 4.** Differences in energy-adjusted intake of total dietary fibre (g/MJ) by age group and socio-demographic factors. Breastfed 1-year-olds excluded from analyses

	1 year		3 years		6 years	
	Mean	sd	Mean	sd	Mean	sd
Sex						
Girl	2·73	0·84	2·37	0·7	2·34	0·64
Boy	2·82	0·91	2·39	0·73	2·3	0·68
*P* value*	0·001		0·245		0·151	
Region						
Tampere University Hospital	2·71	0·87	2·44	0·74	2·38	0·67
Oulu University Hospital	2·87	0·89	2·3	0·66	2·23	0·65
*P* value*	< 0·001		< 0·001		< 0·001	
Mother’s age (years)						
< 25	2·69	0·86	2·28	0·69	2·22	0·64
25–29	2·81	0·87	2·37	0·69	2·35	0·66
30–34	2·82	0·88	2·4	0·72	2·33	0·67
≥ 35	2·75	0·93	2·46	0·76	2·33	0·68
*P* value†	0·01		< 0·001		0·029	
Father’s age (years)						
< 25	2·74	0·93	2·23	0·64	2·26	0·6
25–29	2·8	0·86	2·37	0·69	2·31	0·66
30–34	2·78	0·87	2·39	0·72	2·3	0·66
≥ 35	2·77	0·9	2·43	0·76	2·36	0·68
* P* value†	0·748		< 0·001		0·205	
Mother’s education						
Basic	2·56	0·82	2·2	0·7	2·28	0·67
Vocational	2·65	0·87	2·22	0·67	2·16	0·61
Secondary vocational	2·83	0·86	2·4	0·7	2·32	0·66
Academic	2·9	0·93	2·55	0·73	2·5	0·68
* P* value†	< 0·001		< 0·001		< 0·001	
Father’s education						
Basic	2·55	0·74	2·23	0·63	2·19	0·61
Vocational	2·7	0·86	2·28	0·71	2·23	0·63
Secondary vocational	2·83	0·92	2·43	0·71	2·38	0·66
Academic	2·92	0·88	2·53	0·74	2·44	0·7
*P* value†	< 0·001		< 0·001		< 0·001	
Highest education of the parents						
Basic	2·56	0·66	2·23	0·66	2·45	0·79
Vocational	2·63	0·86	2·2	0·67	2·14	0·61
Secondary vocational	2·78	0·86	2·37	0·7	2·31	0·64
Academic	2·91	0·91	2·52	0·73	2·46	0·69
*P* value†	< 0·001		< 0·001		< 0·001	
Number of siblings						
0	2·8	0·9	2·45	0·73	2·35	0·68
1	2·73	0·85	2·32	0·68	2·32	0·63
≥ 2	2·79	0·88	2·3	0·7	2·24	0·64
* P* value†	0·094		< 0·001		0·015	
Mother’s smoking during pregnancy						
Non-smoking	2·81	0·9	2·4	0·72	2·33	0·67
Smoking	2·55	0·75	2·15	0·65	2·09	0·57
*P* value*	< 0·001		< 0·001		< 0·001	

* Independent samples *t* test.

† Univariate analysis of variance.

### Food sources of total dietary fibre and dietary fibre fractions

**Fig. 1.**
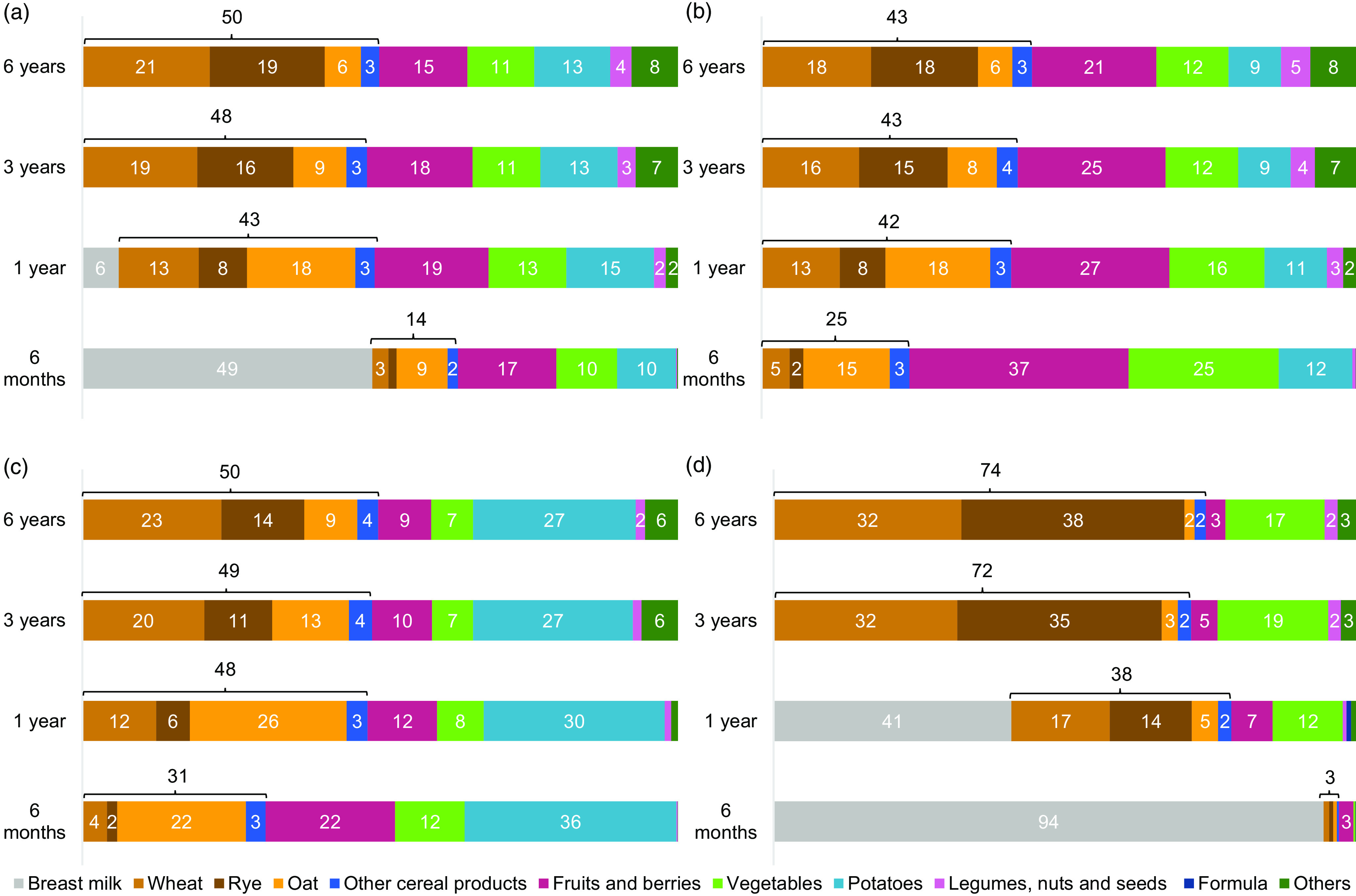
Food sources of (a) total dietary fibre, (b) insoluble dietary fibre, (c) dietary fibre soluble in water but insoluble in 76 % aqueous ethanol and (d) dietary fibre soluble in water and soluble in 76 % aqueous ethanol (SDFS). Percentages under 2 not shown. 6 months *n* 4886, 1 year *n* 4416, 3 years *n* 3463, 6 years *n* 2349.

The food sources of total dietary fibre and dietary fibre fractions are presented in Fig. 1. The order of importance of different food sources resembled each other in 1-, 3-, and 6-year-olds, while often distinctly different in 6-month-olds. For 1-, 3-, and 6-year-olds, cereal products were the main source of total dietary fibre as well as all dietary fibre fractions, except SDFS in 1-year-olds. For total dietary fibre, cereal products were followed by fruits and berries, potatoes, and vegetables in these age groups. For IDF, fruits and berries were second, vegetables third, and potatoes the fourth most important sources. For SDFP, cereal products were followed by potatoes, and fruits and berries and vegetables played more minor roles. For SDFS, the primary source of 1-year-olds was breast milk, whereas for 3- and 6-year-olds vegetables followed cereal products, and the role of fruits and berries was less pronounced.

At 6 months, breast milk was the main source of total dietary fibre, and SDFS almost exclusively came from breast milk. As SDFS is the only fibre fraction present in breast milk (due to HMO) it did not contribute to IDF or SDFP. Instead, fruits and berries were the most important source of IDF, and potatoes were the primary source of SDFP.

Further examination of the cereal product sources of dietary fibre revealed a shift in the role of the different grains with the age of the children: oat was a more important source of dietary fibre for the younger children, while the proportions of wheat and rye increased with age. Rye was a distinct source of SDFS, in contrast to its less prominent role as a source of the other dietary fibre fractions.

## Discussion

The present study describes intake and sources of total dietary fibre and dietary fibre fractions in children aged 6 months, 1 year, 3 years, and 6 years. Absolute intake of dietary fibre increased with age, but energy adjusted intake decreased with age. Breastfeeding was a major determinant of dietary fibre intake, contributing to higher total dietary fibre intake and a higher proportion of dietary fibre coming from the SDFS fraction. This was especially clear in 6-month-olds, where breast milk derived SDFS accounted for almost half of total dietary fibre intake. In older and non-breastfed children, insoluble dietary fibre was the major dietary fibre fraction, followed by the soluble dietary fibre fractions SDFP and SDFS. Important food sources of dietary fibre included cereal products, fruits and berries, potatoes, and vegetables, while the role of legumes, nuts, and seeds was relatively minor. We also studied socio-demographic differences in energy-adjusted intake of total dietary fibre in non-breastfed 1-year-olds, 3-year-olds, and 6-year-olds. Energy-adjusted intake of total dietary fibre was higher in children with older, more highly educated parents, and lower in those with smoking mothers.

Strengths of our study include utilising a representative, large sample with balanced drop-out, and follow-up through formative years of a child’s diet. Although prospective, self-reported dietary assessment methods may cause conscious or unconscious changes in food behaviour, our dietary data has been found to be of high quality in previous validation studies^(25,26)^. Our study is among the first conducted using new, CODEX-compliant values for dietary fibre, and provides novel information on intake and sources of both total dietary fibre as well as dietary fibre fractions. To our knowledge, this is the first study assessing the role of HMO to dietary fibre intake in relation to the child’s total diet.

Regarding the weaknesses of the study, we must consider that dietary fibre fractions are merely an analytical classification and do not necessarily represent the diverse nature of dietary fibre accurately in terms of physiological effects. Furthermore, not all our updated database values were based on directly AOAC 2011·25 analysed values. This would, however, be unfeasible considering the number of entries in the database, as well as the fact that new, more refined dietary fibre analytical methods are still being developed^(27,28)^. In the future, the methods AOAC 2009·01 and 2011·25 will be replaced by the method AOAC 2017·16, as it is still a further improved version of the previous one. Due to the changes, the latest method better mimics physiological conditions and improves the analysis of resistant starch and some NDO. The scale of effects on dietary fibre results are however likely to be much smaller than the effect of the transition from older methods to the CODEX-compliant methods.

The levels of total dietary fibre intake of Finnish children are in line with previous results of children in selected countries^(6)^. We did find very high energy-adjusted levels of dietary fibre intake in breastfed children, but there is no previous data on dietary fibre intake in breastfed children that would have included HMO. Based on previous literature updated dietary fibre analysis methods also produce higher dietary fibre values^(21,22,29,30)^. Due to the introduction of a new definition and analytical methods, it may be necessary to re-evaluate dietary fibre intake recommendations, as updates may result in higher intakes than previously produced.

Dietary fibre density of the diet decreasing as the child adapts a diet more similar to the rest of the family has also been reported in German children^(31)^. It is possible that parents are more mindful of the healthiness of their child’s diet when preparing food separately and adopting a diet more similar to the rest of the family lead to a decrease in nutrient and dietary fibre density. Also consistent with our findings, USA children within the lowest quartile of dietary fibre intake had less educated caregivers with less purchasing power compared with children in the highest quartile^(32)^. Other nutrients were not considered in our study but based on the sources of dietary fibre in our study children, a higher intake of dietary fibre could in general reflect a more nutrient-dense diet.

In source analyses, we did not find specific dietary fibre fractions to come from specific food groups. This would imply that source classifications such as cereal fibre or fruit fibre do not represent an analytically distinguishable dietary fibre type. Dietary fibre fraction analysis could provide an alternative classification method for studying health effects of different types of dietary fibre with reduced confounding effects by the food matrix.

Breast milk provides an infant with human milk oligosaccharides, which are vital for intestinal colonisation by gut microbiota, maturation of the immune system and in providing protection from infection^(33,34)^. Mechanisms include providing structurally diverse energy sources for the microbiota, inhibition of microbial adhesion, cell signalling, short-chain fatty acid production and anti-inflammatory properties^(33,34)^. Fructo- and galacto-oligosaccharides can be added to infant formulas to mimic the prebiotic content of breast milk, but based on our results, formulas can in no way replace breast milk as a source of NDO. More research is needed on HMO and the age and order of introduction to solid foods in relation to long-term child health.

Dysbiosis of the gut microbiota has been associated with the development of autoimmune diseases such as type 1 diabetes, and it is possible that appropriate prebiotic dietary fibre intake may ameliorate these processes^(35)^. However, what exactly is appropriate remains unclear, as higher intake of total dietary fibre has also been associated with increased risk of type 1 diabetes^(36)^. This could, however, be due to contaminants in the fibre matrix rather than dietary fibre itself, as harmful associations of, e.g. trypsin inhibitors, mycotoxins, heavy metals, or pesticides, all common contaminants in the cereal fibre matrix, have been speculated^(36)^.

Cereal products, the main source of dietary fibre in our study children aged 1, 3 and 6 years, are also sources of harmful mycotoxins, inorganic arsenic, cadmium and lead^(37,38)^. While exposure to these substances does not seem to be an issue in adults, recent data indicate that safety levels may be exceeded in children. As much as 88 % of Finnish 3-year-olds and 64 % of Finnish 6-year-olds exceed the tolerable weekly intake of cadmium^(38)^. Diversifying dietary fibre sources in children could therefore be called for. Based on our results, there would be room for increasing the role of legumes, nuts and seeds in the diet of Finnish children.

Current recommendations for dietary fibre intake in children are inconsistent^(39–45)^ as well as loosely evidence based. Recommendations for dietary fibre intake in children are often based on extrapolation from adults, but children are not ‘little adults’ and dietary fibre may serve a multitude of health benefits in children beyond laxation^(46)^. More research is needed to assess health effects of dietary fibre and dietary fibre fractions in children, especially young children with developing gut microbiota and immunity. The role of the different dietary fibre fractions on the development of gut microbiota, gut immunity and childhood-onset autoimmune diseases should be explored. New recommendations for intake of dietary fibre for children should consider the multifactorial nature of dietary fibre for children’s health.

The Finnish nutrition recommendations for infants from 2004^(47)^, around the time of the collection of our data, recommended exclusive breast-feeding until the age of 6 months. Introduction of solid foods was recommended to be started with spoon-fed purees made from potatoes, vegetables, fruits and berries. Cereal products, in the form of spoon-fed porridge, were to be added only after these introductory foods. The recommendations were reflected in our results: due to their later introduction, cereal products were a distinctly less important source of dietary fibre in children aged 6 months than at later age points. Also, a shift from oat as a more important source of dietary fibre in younger children to rye and wheat gaining importance in older children could be seen, as porridge is typically made from oat, but bread more commonly from wheat and rye in Finland. These food cultural phenomena could imply that our results can only be generalised to populations with similar food cultures as well as breast-feeding and early feeding recommendation.

The generalisability of our results may be restricted if other food composition databases are not updated with appropriate AOAC-method-based values as well. With the establishment of a CODEX definition for dietary fibre, homogenous analysis methods should allow different countries to utilise analysis results across borders. European food composition databases alone contain over 39 000 food entries, creating strong incentive for sharing the financial burden of database updating. Usage of outdated definitions and analytical methods not only hampers the exchange of database entries but also the comparability of study results. Mismatched dietary fibre definitions are an additional confounder that should be considered when studying dietary fibre, as the definition and analytical methods used can affect results related to the dietary fibre content and composition of foods. In young children, special consideration should be given to the inclusion of NDO and especially HMO in the definition of dietary fibre.

### Conclusions

Our study based on updated CODEX-compliant food database values for dietary fibre and dietary fibre fractions found that the intake of dietary fibre fractions as well as the contribution of different food sources to dietary fibre intake shift through the first 6 years of life. Including oligosaccharides in the definition of dietary fibre values results in high intakes of total dietary fibre and SDFS in breastfed children, as breast milk is a major source of non-digestible oligosaccharides. In non-breastfed children, IDF is the major dietary fibre fraction followed by SDFP and SDFS, with cereal products, fruits and berries, potatoes and vegetables acting as the major food sources of dietary fibre.
